# Dr. C. Schuhart

**Published:** 1917-12

**Authors:** 


**Dr. C. Schuhart,** P. & S., Baltimore, 1881, died at his home in Rochester, Sept. 23, aged 62.

